# Real-World Effectiveness and Patient Stratification for Vedolizumab Treatment in Crohn’s Disease: A Multicenter Retrospective Study

**DOI:** 10.1093/gastro/goaf096

**Published:** 2025-10-31

**Authors:** Kang Chao, Zhaopeng Huang, Hongsheng Yang, Yun Qiu, Lingya Yao, Ren Mao, Jing Liu, Qian Cao, Minhu Chen, Xiang Gao

**Affiliations:** Department of Gastroenterology, The Sixth Affiliated Hospital, Sun Yat-sen University, Guangzhou, Guangdong, P. R. China; Biomedical Innovation Center, The Sixth Affiliated Hospital, Sun Yat-sen University, Guangzhou, Guangdong, P. R. China; Department of Gastroenterology, The Sixth Affiliated Hospital, Sun Yat-sen University, Guangzhou, Guangdong, P. R. China; Biomedical Innovation Center, The Sixth Affiliated Hospital, Sun Yat-sen University, Guangzhou, Guangdong, P. R. China; Department of Gastroenterology, The Sixth Affiliated Hospital, Sun Yat-sen University, Guangzhou, Guangdong, P. R. China; Biomedical Innovation Center, The Sixth Affiliated Hospital, Sun Yat-sen University, Guangzhou, Guangdong, P. R. China; Department of Gastroenterology, The First Affiliated Hospital, Sun Yat-sen University, Guangzhou, Guangdong, P. R. China; Sir Run Run Shaw Hospital, Zhejiang University School of Medicine, Hangzhou, Zhejiang, P. R. China; Department of Gastroenterology, The First Affiliated Hospital, Sun Yat-sen University, Guangzhou, Guangdong, P. R. China; Sir Run Run Shaw Hospital, Zhejiang University School of Medicine, Hangzhou, Zhejiang, P. R. China; Sir Run Run Shaw Hospital, Zhejiang University School of Medicine, Hangzhou, Zhejiang, P. R. China; Department of Gastroenterology, The First Affiliated Hospital, Sun Yat-sen University, Guangzhou, Guangdong, P. R. China; Department of Gastroenterology, The Sixth Affiliated Hospital, Sun Yat-sen University, Guangzhou, Guangdong, P. R. China; Biomedical Innovation Center, The Sixth Affiliated Hospital, Sun Yat-sen University, Guangzhou, Guangdong, P. R. China

**Keywords:** vedolizumab, Crohn’s disease, effectiveness, patient stratification

## Abstract

**Background:**

Although selecting the appropriate patients for vedolizumab (VDZ) treatment was challenging, this multicenter, retrospective study evaluated the real-world effectiveness of VDZ and identified the patients who would benefit from VDZ therapy.

**Methods:**

A total of 264 patients from three tertiary care centers specializing in inflammatory bowel disease were treated with VDZ. The outcomes assessed included steroid-free remission, clinical remission, objective response, and remission at Weeks 26 and 52. Least Absolute Shrinkage and Selection Operator regression and multivariate analyses were performed to identify independent predictors, and a nomogram was developed to predict steroid-free remission at Week 26.

**Results:**

The rates of steroid-free remission and clinical remission were 46.6% and 47.0% at Week 26, and both were 38.6% at Week 52. Objective response and remission were achieved in 41.5% and 14.8% of patients at Week 26, compared with 20.7% and 11.4% at Week 52. Bio-naïve patients without active intestinal fistula, and with low inflammation burden (Crohn’s Disease Activity Index ≤ 220 and C-reactive protein ≤ 10 mg/L) showed the highest rates of steroid-free remission and objective remission at both time points (all *P *< 0.05), along with a superior therapeutic continuation (*P *< 0.001). The nomogram, incorporating these factors, effectively predicted steroid-free remission at Week 26 (area under the curve = 0.830) and Week 52 (area under the curve = 0.702). VDZ was well tolerated with an adverse reaction rate of 4.2% and no serious adverse events.

**Conclusions:**

VDZ was effective and safe in treating Crohn’s disease. Patients who were bio-naïve, without active intestinal fistulas, and who had milder baseline disease activity were more likely to benefit from VDZ therapy.

## Introduction

Crohn’s disease (CD) is a chronic, non-specific inflammatory intestinal disease, with the main clinical manifestations of abdominal pain and diarrhea. If not treated promptly, it may lead to complications such as intestinal fistula and abdominal abscesses, placing a significant burden on the medical community [1,2].

In the era of biologics, more treatment selections such as vedolizumab (VDZ) are available for CD [3,4]. VDZ is a recombinant humanized IgG1 monoclonal antibody that specifically binds to α4β7 integrin. As such, it effectively blocks T-lymphocyte trafficking into the intestine and regulates intestinal inflammation [5]. Additionally, VDZ is effective in inducing and maintaining remission of CD. In support of this, the GEMINI trial showed that 31.4% was achieved in clinical response at Week 6, and 39.0% was achieved in clinical remission at Week 52 [6]. Furthermore, sustained clinical remission in long-term follow-up has been shown in open-label extension data from the GEMINI LTS. Notably, the study showed that 83% and 89% of patients were in remission after 104 and 152 weeks, respectively [7].

Unfortunately, there is still a considerable proportion of patients who have lost response to VDZ. No single factor can accurately predict who will respond quickly to VDZ therapy or will benefit from therapeutic drug monitoring or dose intensification [8]. Accurate identification of patients with a high probability response to VDZ will allow a personalized medicine approach to increase efficacy. Dulai *et al.* developed and validated a clinical decision support tool (CDST) that classified CD patients into low, intermediate, and high probability of response to VDZ based on a scoring system [9]. However, it is worth noting that the patients included in this scoring system were selected based on strict criteria in Phase III clinical trials. Thus, the scoring system may not accurately predict the real-world effectiveness [6,10]. Therefore, we conducted a multicenter, retrospective, real-world study to explore the clinical and objective effectiveness of VDZ in CD patients. Additionally, we aimed to identify the optimal populations most likely to benefit from this treatment in a real-world setting.

## Methods

### Study design

This was a multicenter, retrospective, observational real-world cohort study. Patients were enrolled in three tertiary inflammatory bowel disease centers in China: the Sixth Affiliated Hospital, Sun Yat-sen University, Guangzhou, P. R. China; SIR Run Run Shaw Hospital of Zhejiang University School of Medicine, Hangzhou, P. R. China; and the First Affiliated Hospital, Sun Yat-sen University, Guangzhou, P. R. China. CD patients receiving VDZ between May 2020 and December 2024 were considered for inclusion.

This study was approved by the Ethics Committee of the Sixth Affiliated Hospital, Sun Yat-sen University (approval number: 2022ZSLYEC-326) and subsequently by all the other participating centers.

### Patients

Inclusion criteria were as follows: (i) adult patients (≥18 years old) with a confirmed diagnosis of CD based on the current guideline [11,12], and (ii) patients received at least one dose of VDZ. Exclusion criteria included as follows: (i) patients had a definitive or transitory stoma, or (ii) patients had insufficient clinical data to evaluate the effectiveness at Week 26.

### Data collection

In our routine clinical practice, patients were scheduled to evaluate the clinical and objective outcomes at Weeks 26 and 52. Endoscopy, intestinal ultrasound, computed tomography enterography, or magnetic resonance enterography were also conducted simultaneously. Baseline and follow-up data of patients were collected from electronic medical records. Two physicians at each center were tasked with data retrieval. One extracted the data independently using a standardized form, while the other verified it. Communication among investigators was conducted through online conferences, with disagreements resolved through discussion to reach a consensus. Data were collected, including sex, age, smoking status at baseline, disease duration, symptoms, history of bowel surgery, disease location and behavior by Montreal classification, disease activity based on Crohn’s Disease Activity Index (CDAI), medication history, endoscopic manifestations, intestinal ultrasound, computed tomography enterography or magnetic resonance enterography manifestations, laboratory parameters, therapeutic regimens, and adverse events.

### Investigated drug

VDZ 300 mg was administered by intravenous injection at Week 0, Week 2, and Week 6 for induction. Additionally, these were followed by every 8 weeks as maintenance. Patients with inadequate response or loss of response to the standard dose of VDZ could undergo therapy adjustment, including dose escalation, concomitant other therapies, or treatment conversion. Notably, these adjustments were made at the discretion of the physician, and the options involved a shared decision-making process between the physician and the patient. For VDZ dose escalation, options included re-induction with intravenous administration or shortening the dosing interval to 4–6 weeks. Concomitant steroid use was allowed per the physician’s judgment. The standardized tapering protocol-initiated prednisone at 0.75–1 mg/kg/day (max 60 mg/day), with dose reduction commencing upon clinical improvement (typically within 2–4 weeks). Furthermore, tapering occurred at 5 mg/week until reaching 50% of the initial dose, then slowed to 5 mg every 2 weeks until discontinuation.

### Outcomes and definitions

The primary outcome was the rate of steroid-free remission at Weeks 26 and 52. Secondary outcomes included clinical remission, objective response, and remission at Weeks 26 and 52. Other secondary measures included primary non-response, secondary loss of response, and adverse events during follow-up. Additionally, predictors of steroid-free remission at Week 26 were analyzed, and a nomogram model was constructed to enable patient stratification for VDZ treatment.

A CDAI score was used to evaluate the clinical effectiveness of VDZ treatment in CD patients [13]. The disease was considered mildly active if CDAI was 150–220, moderately active if CDAI was 221–450, and severely active if CDAI > 450. Steroid-free remission was defined as CDAI < 150 for CD patients with no concomitant steroids. Clinical relapse was defined as either CDAI ≥ 150 with a ≥ 70-point increase from baseline, or definitive clinical relapse requiring immediate intervention per treating physician assessment. Primary non-response was defined as failure to achieve ≥ 70-point CDAI reduction in moderate-severe baseline disease (CDAI > 220), failure to attain remission (CDAI < 150) in mild/remission baseline disease, or requiring escalation/rescue therapy per physician judgment. Secondary loss of response was defined as recurrence of active disease (CDAI > 150) following initial clinical remission, failure to maintain ≥ 70-point CDAI reduction from baseline, or requirement for escalation/rescue therapy per physician assessment. Objective effectiveness was defined based on results obtained from at least one objective technique, including endoscopy, bowel ultrasound, and computed tomography enterography or magnetic resonance enterography. The definitions of objective response and remission were clinically relevant for the real-world setting and were based on endpoints recommended by STRIDE-II [14–16]. Endoscopic efficacy was evaluated using the simplified endoscopic score for Crohn’s disease (SES-CD). Endoscopic response was defined as a reduction in SES-CD of ≥ 50% from baseline. Endoscopic remission was defined as SES-CD ≤ 2 points after treatment [17]. Ultrasonic response was defined as a reduction of bowel wall thickness (BWT) evaluated by bowel ultrasound; ultrasound remission was defined as a normal BWT (≤ 3 mm for the small bowel and ≤ 4 mm for the colon) without any detectable complication [18]. Radiological response was defined by improvement of BWT, inflammatory fat, mural blood flow, and hyperenhancement, evaluated through computed tomography or magnetic resonance enteroclysis. Radiological remission was defined as normalization of these inflammatory parameters [19,20].

Therapy continuation was defined as maintenance of VDZ treatment per protocol without permanent discontinuation. Discontinuation criteria included: lack or loss of response, adverse events, non-adherence (≥ 2 consecutive missed doses), and patient preference. The total follow-up time was calculated from the date of the first dose of VDZ until the last date of administration or discontinuation. To assess the proportion of patients achieving clinical and objective responses or remission at Weeks 26 and 52, those who failed treatment with VDZ and subsequently discontinued therapy were classified as non-responders in both clinical and objective evaluations. Additionally, patients who responded to the treatment but did not have sufficient follow-up time were excluded from the calculation of the overall response or remission rate at specific time points.

### Statistical analysis

All statistical analyses were conducted using R software (version 4.4.2; R Foundation) and SPSS 26.0 (IBM Corp.). Continuous variables were presented as mean ± standard deviation for normally distributed data or median with interquartile range for non-normal distributions. Categorical variables were expressed as frequencies with percentages. Intergroup comparisons were performed using χ^2^ tests or Fisher’s exact tests for proportions. In contrast, Student’s *t*-tests or Mann-Whitney *U* tests were applied for continuous variables based on normal distribution. Treatment persistence was evaluated through Kaplan-Meier survival analysis, with between-group differences assessed using log-rank tests.

Variable selection was initially performed through Least Absolute Shrinkage and Selection Operator (LASSO) regression analysis using the “glmnet” package in R. A 10-fold cross-validation process was implemented to identify the optimal regularization parameter (λ), with lambda.min selected to maximize model sensitivity while maintaining predictive performance. The variables chosen through LASSO regression were further analyzed using multivariate logistic regression to determine the final independent predictors. Variables with a *P*-value < 0.05 in the multivariate analysis were retained for the final predictive model. Based on these independent predictors, a logistic regression model was constructed and visually presented as a nomogram to facilitate clinical interpretation. The Receiver operating curve was applied to evaluate the discriminative ability of the nomogram model, with the accuracy assessed by the area under the curve (AUC). Additionally, calibration curves and decision curve analysis were performed in the study.

All statistical tests were two-sided. A *P*-value of less than 0.05 was considered statistically significant.

## Results

### Study population

**Figure 1.**
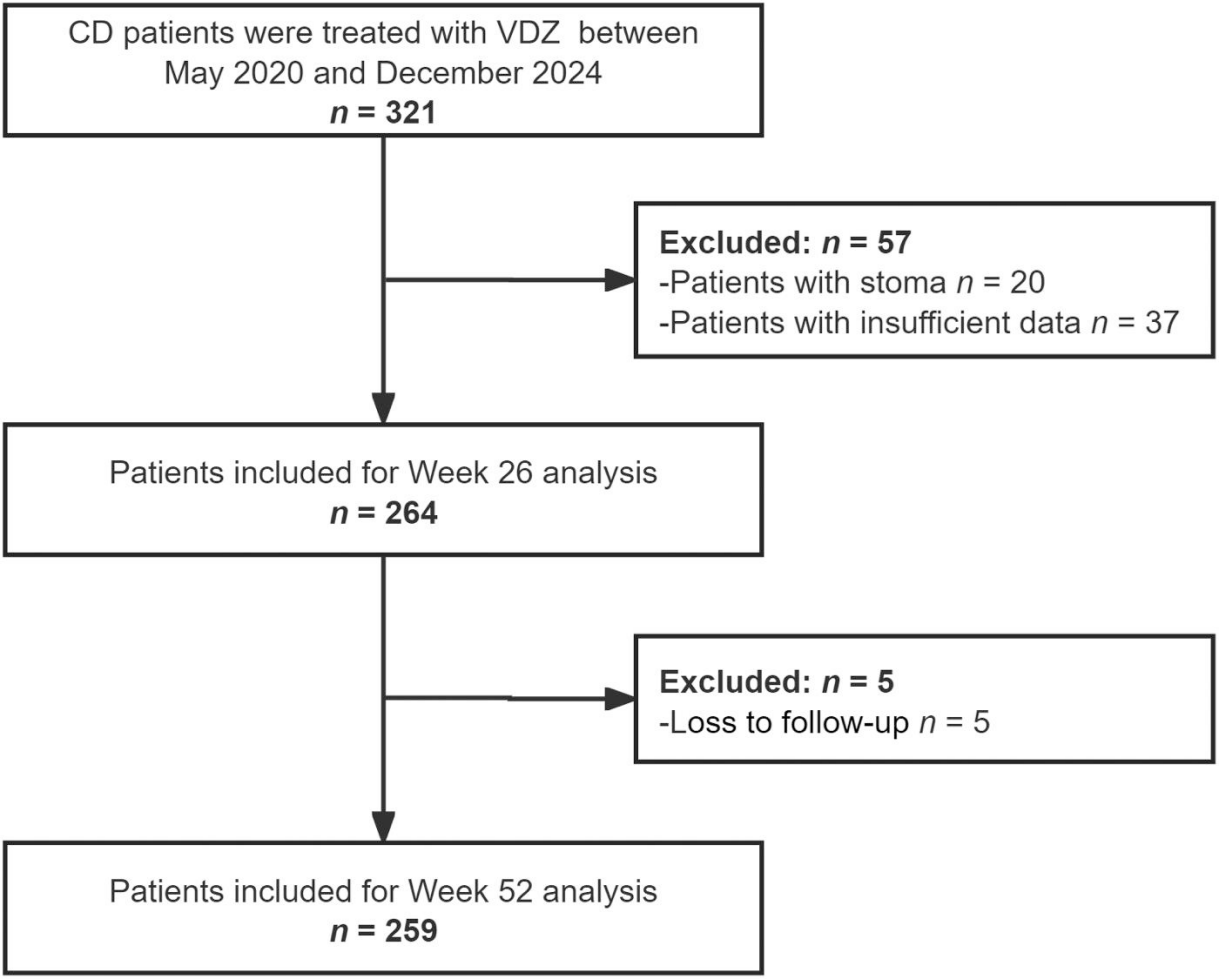
Flow chart illustrating the selection of patients in this study. VDZ = vedolizumab; CD = Crohn’s disease.

A total of 321 patients who received VDZ treatment between May 2020 and December 2024 were considered for inclusion, with 264 patients ultimately meeting the criteria for participation (Figure 1). At baseline, 175 patients (66.3%) were in the active stage, while 89 patients (33.7%) were in remission. The reasons for administering VDZ treatment during clinical remission included objective active disease (20.1%), sequential therapy after exclusive enteral nutrition- or steroid-induced remission (9.5%), and previous adverse drug reactions (4.2%). The baseline characteristics of patients are shown in Table 1.

**Table 1.** Baseline data of vedolizumab-treating patients with Crohn’s disease.

Characteristics	*n *= 264
Male, *n* (%)	161 (61.0)
Age, years, median (IQR)	32.0 (25.0, 40.8)
Disease duration, months, median (IQR)	57.5 (24.0, 96.0)
Active smokers, *n* (%)	25 (9.5)
Previous bowel resection, *n* (%)	92 (34.8)
Location classified by Montreal Classification, *n* (%)	
L1	47 (17.8)
L2	8 (3.0)
L3	209 (79.2)
L4	22 (8.3)
Behavior classified by Montreal Classification, *n* (%)	
B1	109 (41.3)
B2	78 (29.5)
B3	77 (29.2)
Active intestinal fistula, *n* (%)	40 (15.2)
Entero-enteric	39 (14.8)
Enterocutaneous	1 (0.4)
Perianal disease, *n* (%)	137 (51.9)
Bio-naive, *n* (%)	124 (47.0)
Previous EEN therapy, *n* (%)	107 (40.5)
Previous steroids therapy, *n* (%)	95 (36.0)
Previous immunomodulator therapy, *n* (%)	158 (59.8)
CDAI at baseline, median (IQR)	191.0 (114.1, 243.5)
CDAI ≥ 150, *n* (%)	175 (66.3)
CDAI > 220, *n* (%)	96 (36.4)
Reasons for administering VDZ treatment in patients with clinical remission at baseline, *n* (%)	
Objective active disease	53 (20.1)
Sequential therapy after EEN- or steroid-induced remission	25 (9.5)
Previous adverse drug reactions	11 (4.2)
Use of steroids at baseline, *n* (%)	22 (8.3)
EEN at baseline, *n* (%)	28 (10.6)
CRP, mg/L, median (IQR)	4.6 (1.4, 18.3)
CRP > 10 mg/L, *n* (%)	95 (36.0)
ALB, g/L, median (IQR)	38.6 (35.1, 41.8)
ALB < 35 g/L, *n* (%)	64 (24.2)

ALB = albumin, B1 = nonstricturing and nonpenetrating, B2 = stricturing, B3 = penetrating, CDAI = Crohn's disease activity index, VDZ = vedolizumab, CRP = C-reactive protein, EEN = exclusive enteral nutrition, IQR = interquartile range, L1 = terminal ileum, L2 = colon, L3 = ileocolon, L4 = upper gastrointestinal tract.

### Clinical and objective effectiveness in CD

**Figure 2.**
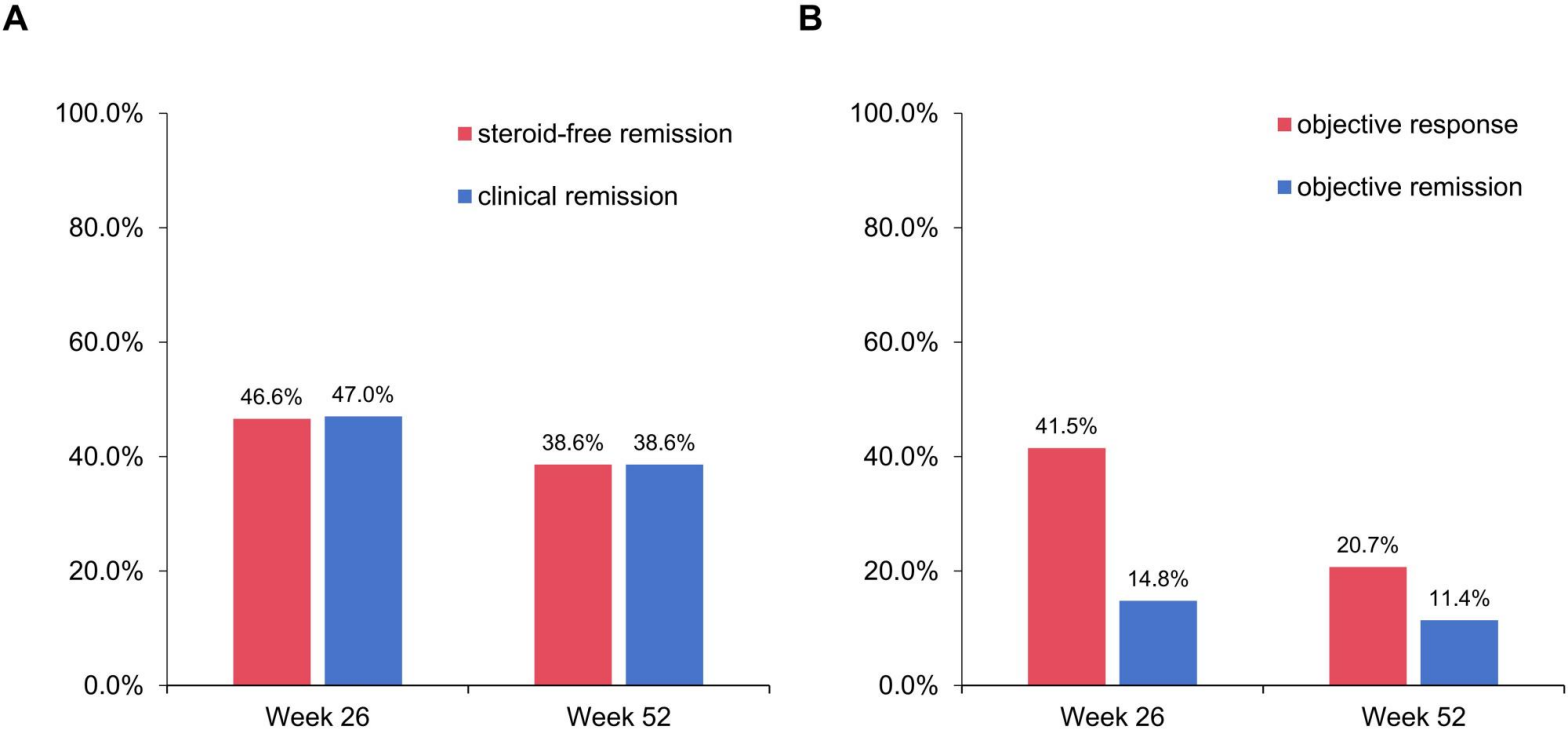
Effectiveness of vedolizumab in treating patients with Crohn’s disease. (A) Steroid-free remission and clinical remission at Weeks 26 and 52. (B) Objective response and remission at Weeks 26 and 52.

In the overall cohort, steroid-free remission and clinical remission were achieved in 46.6% (123 of 264) and 47.0% (124 of 264) of patients at Week 26, respectively; both were 38.6% (100 of 259) at Week 52 (Figure 2A). Among patients with clinically active disease at baseline, the rates of clinical remission and steroid-free remission were 32.6% (57 of 175) at Week 26 and 29.7% (51 of 172) at Week 52. Among those in clinical remission at baseline, clinical relapse occurred in 25.8% (23 of 89) of patients at Week 26 and 43.7% (38 of 87) at Week 52.

The rate of primary non-response was 42.4% (112 of 264) at Week 26, while the rate of secondary loss of response was 35.1% (52 of 148) at Week 52. During follow-up, 47 patients (17.8%) underwent therapy escalation. Of these, 36 patients (76.6%) had their dosing interval shortened to every 4–6 weeks, 8 patients (17.0%) received combination therapy with another biologic (infliximab or ustekinumab) or exclusive enteral nutrition, and 3 patients (6.4%) underwent re-induction therapy. The rate of clinical response to dose escalation was 53.2% (25 of 47).

A total of 176 patients underwent at least one objective assessment from Week 0 to Week 26. Among them, 69.9% (123 of 176) underwent endoscopy evaluation, 49.4% (87 of 176) underwent intestinal ultrasound evaluation, and 58.0% (102 of 176) underwent computed tomography enterography or magnetic resonance enterography evaluation. Notably, 19.9% (35 of 176) of patients had all three objective evaluations, and 37.5% (66 of 176) had two evaluations. The rates of objective response and remission at Week 26 were 41.5% (73 of 176) and 14.8% (26 of 176), respectively. A total of 184 patients underwent at least one objective assessment from Week 0 to Week 52. Furthermore, objective response and objective remission were observed in 20.7% (38 of 184) and 11.4% (21 of 184) at Week 52 (Figure 2B).

### Predictors of steroid-free remission at week 26

**Figure 3.**
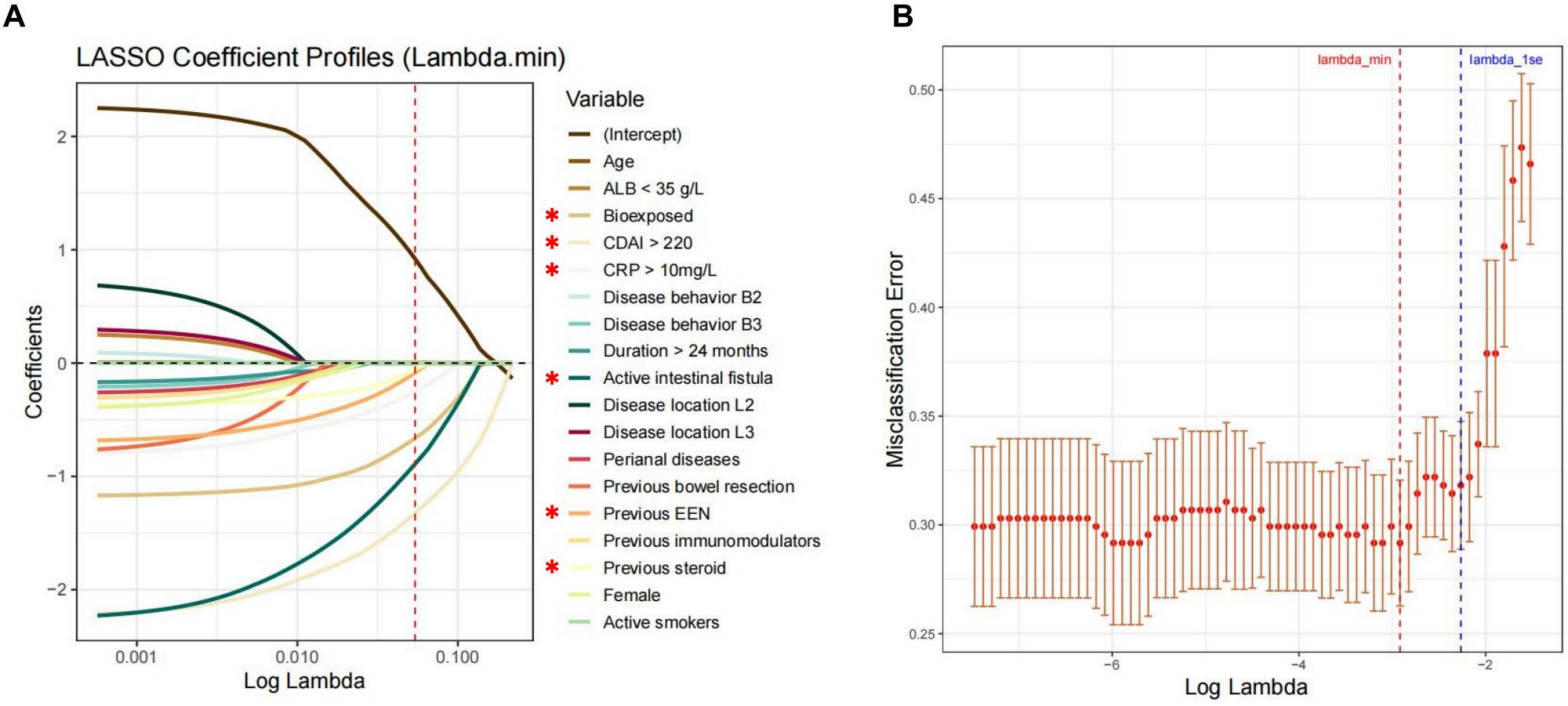
LASSO-Logistic regression. (A) LASSO coefficient profiles of the variables. (B) The cross-validation results. *, The asterisks represent the variables selected by LASSO regression. ALB = albumin; B2 = stricturing; B3 = penetrating; CDAI = Crohn’s disease activity index; CRP = C-reactive protein; EEN = exclusive enteral nutrition; L2 = colon; L3 = ileocolon; LASSO = Least Absolute Shrinkage and Selection Operator.

LASSO regression screened predictors associated with steroid-free remission at Week 26. A 10-fold cross-validation process was implemented to identify the optimal regularization parameter (λ), with λ.min = 0.054 selected to maximize model sensitivity while maintaining predictive performance. The resulting model retained six predictors: active intestinal fistula, bio-exposed, exclusive enteral nutrition-exposed, steroids-exposed, CDAI > 220 points, and baseline C-reactive protein (CRP) > 10 mg/L (Figure 3A and B). These variables were subsequently entered into a multivariate logistic regression to determine the final independent predictors. In the multivariate analyses, the remaining four variables emerged as independent risk factors for failure to achieve steroid-free remission at Week 26: active intestinal fistula at baseline (odds ratio [OR] = 8.345, 95% confidence interval [95% CI] 2.469–28.206, *P *= 0.001), bio-exposed (OR = 3.646, 95%CI 1.925–6.906, *P *< 0.001), CDAI > 220 points (OR = 8.528, 95% CI 4.135–17.591, *P *< 0.001) and CRP > 10 mg/L (OR = 1.969, 95%CI 1.014–3.821, *P *= 0.045). The coefficients for all predictors from the LASSO regression and the multivariate analysis are provided in Supplementary Table 1.

### Probability of patients responding to VDZ stratified by risk factors

**Figure 4.**
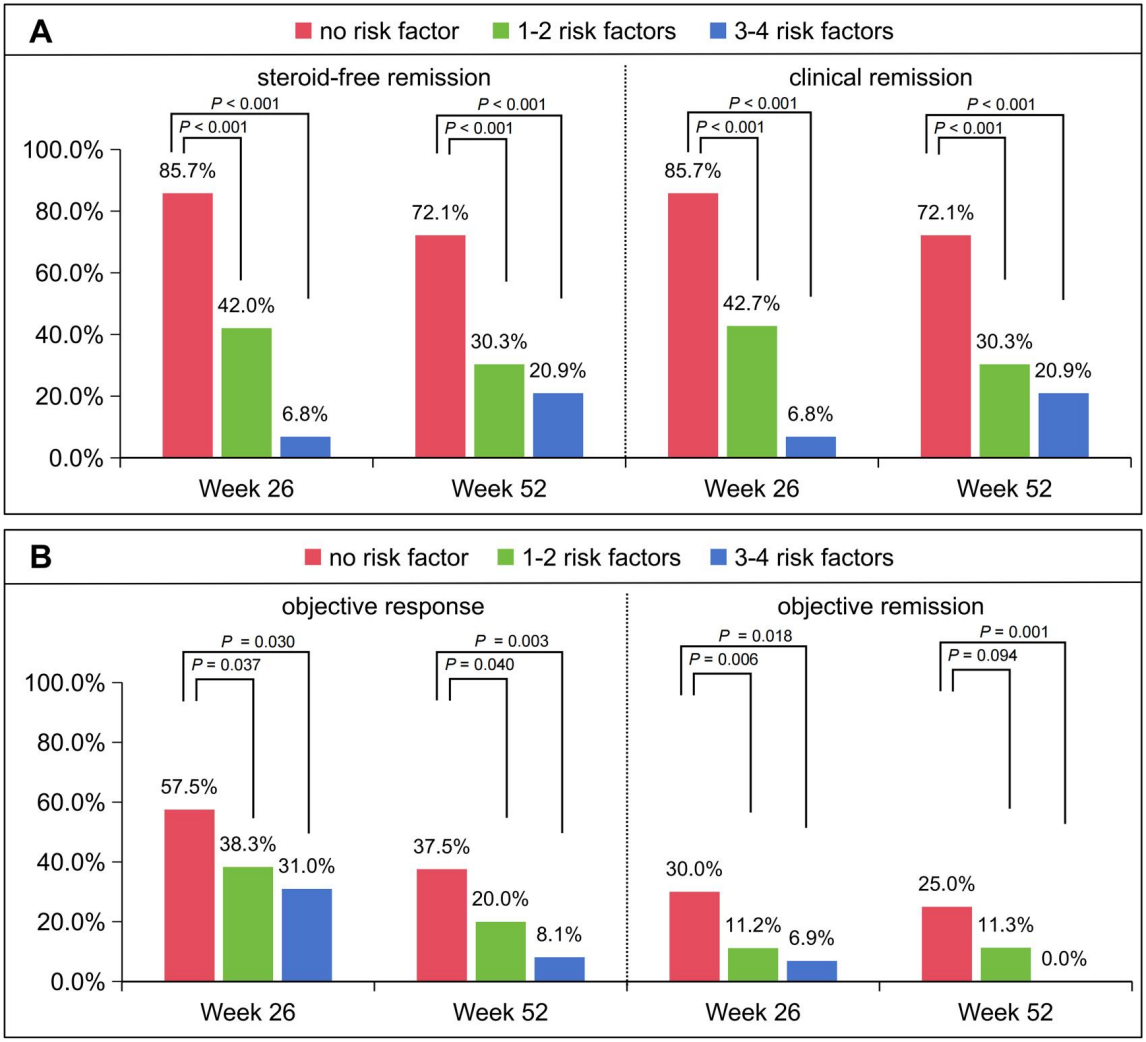
Prognostic stratification of patients in clinical (A) and objective (B) outcome. Risk factors: active fistula, bio-exposed, Crohn’s Disease Activity Index > 220 points and C-reactive protein > 10 mg/L.

**Figure 5.**
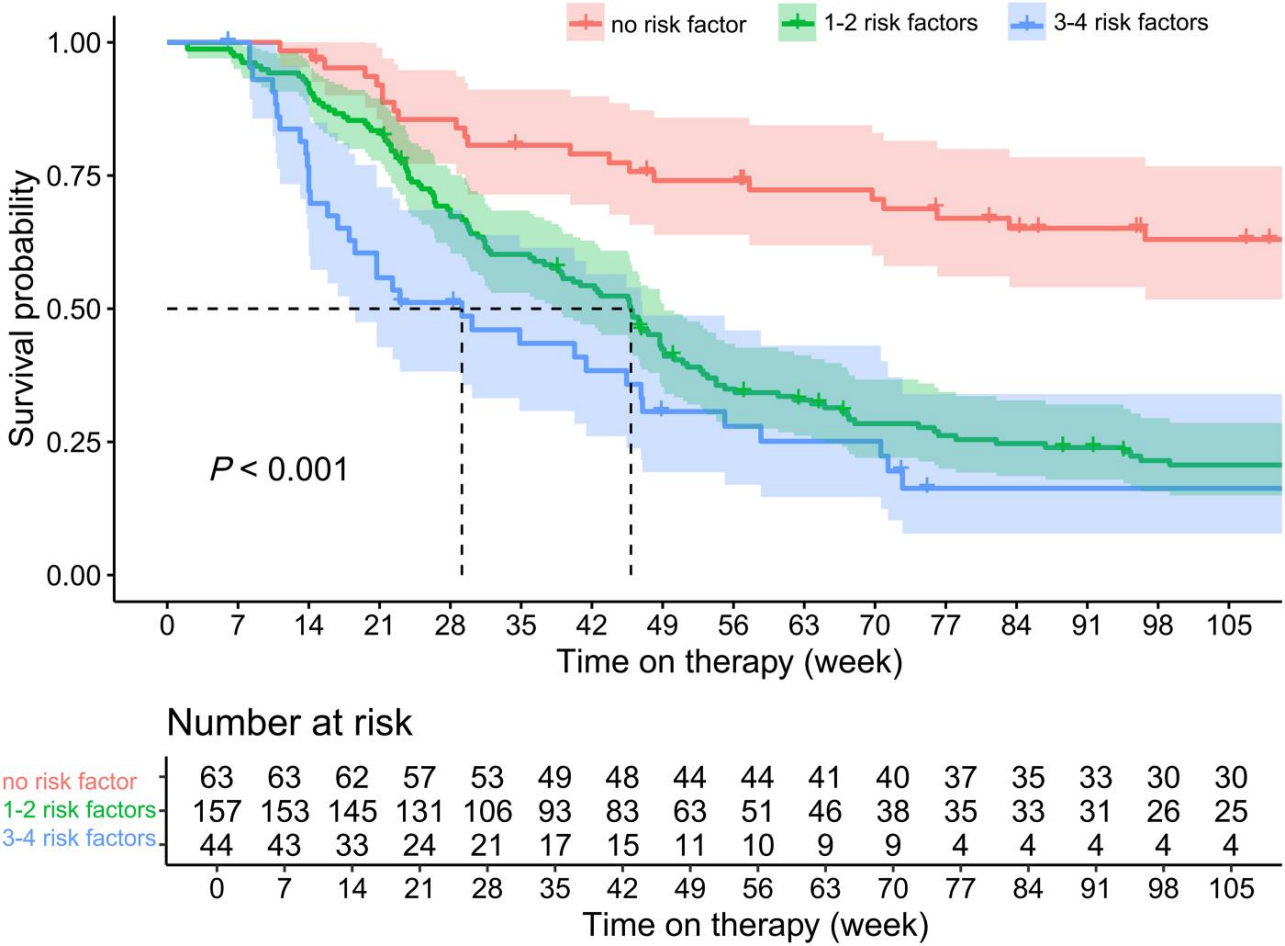
Therapeutic continuation of patients in different risk stratification. Risk factors: active fistula, bio-exposed, Crohn’s Disease Activity Index > 220 points and C-reactive protein > 10 mg/L.

The patient cohort was stratified into distinct risk groups based on the identified risk factors. The low-risk group consisted of patients with no risk factors, the moderate-risk group comprised patients with 1–2 risk factors, and the high-risk group consisted of patients with 3–4 risk factors. Patients in the low-risk group showed the highest rate of steroid-free remission at both Week 26 (85.7% vs 42.0% and 6.8%, all *P *< 0.001) and Week 52 (72.1% vs 30.3% and 20.9%, all *P *< 0.001), when compared with those in the moderate or high-risk group. Additionally, the low-risk group demonstrated the highest rates of objective remission at Week 26 (30.0% vs 11.2% and 6.9%, *P = *0.006 and *P *= 0.018) and Week 52 (25.0% vs 11.3% and 0.0%, *P *= 0.094 and *P *= 0.001) (Figure 4). Similarly, patients in the low-risk group demonstrated superior therapeutic continuation compared with other risk groups (*P *< 0.001) (Figure 5).

### Nomogram model development

**Figure 6.**
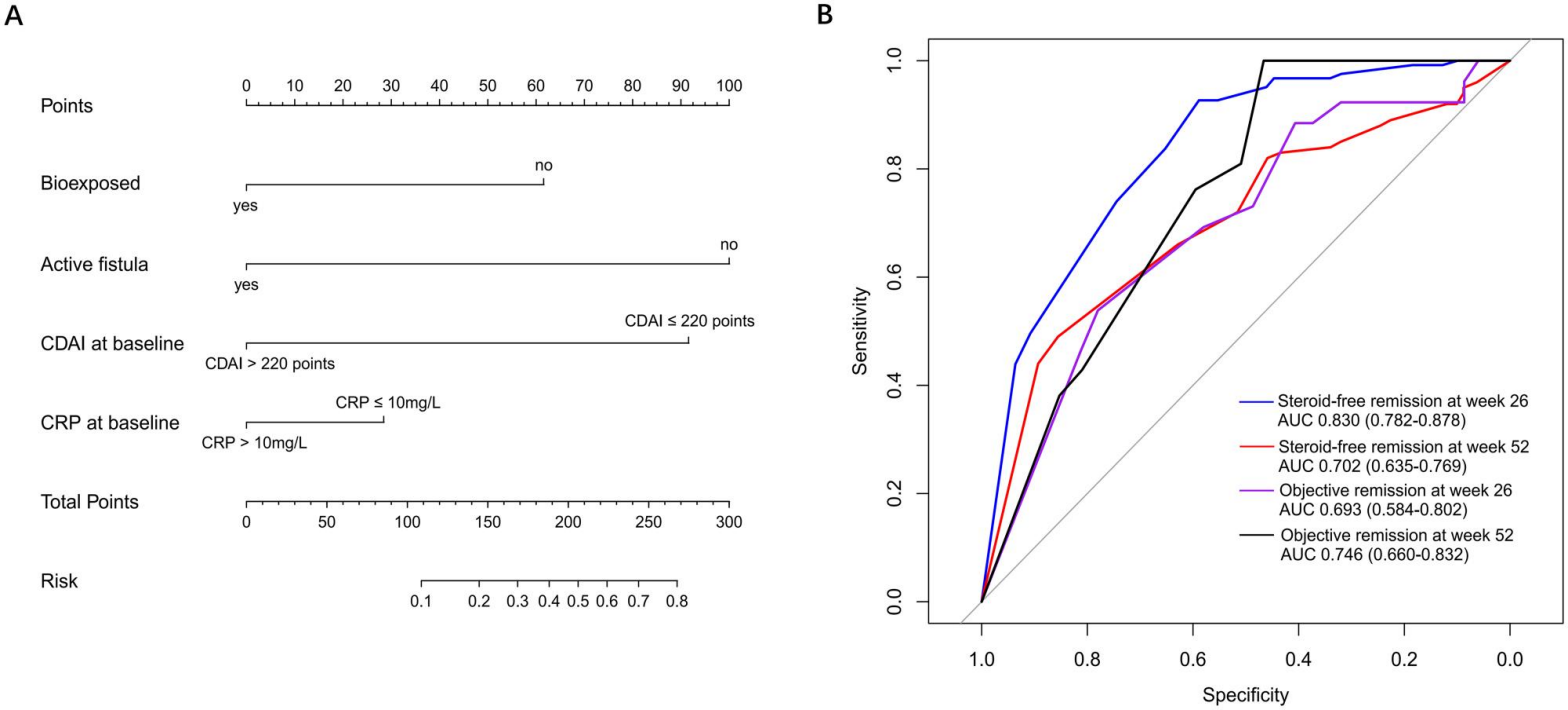
A nomogram model developed by multivariable logistic regression. (A) Nomogram model. (B) ROC of nomogram model in predicting steroid-free remission and objective remission at Weeks 26 and 52. AUC = area under the curve; CDAI = Crohn’s Disease Activity Index; CRP = C-reactive protein; ROC = receiver operating characteristic curve.

A nomogram model was constructed based on the aforementioned four risk factors (Figure 6A). The diagnostic equation was based on multivariable logistic regression analysis as follows: Logit(*P*) = 1.701 + (−2.254 × active fistula) + (−1.388 × bio-exposed) + (−2.064 × CDAI > 220 points) + (−0.641 × CRP > 10 mg/L), where each variable was binary (1 = presence/positive, 0 = absence/negative). The AUC of the nomogram model for predicting steroid-free remission at Week 26 and 52 were 0.830 (0.782–0.878) and 0.702 (0.635–0.769), respectively. For objective remission at Week 26 and 52, the AUC was 0.693 (0.584–0.802) and 0.746 (0.660–0.832), respectively (Figure 6B). Calibration curves and decision curve analysis are shown in Supplementary Figure 1.

### Safety

VDZ demonstrated a generally favorable safety and tolerability profile. A total of 11 patients (4.2%) experienced adverse events. Among these, 3 patients (1.1%) developed symptomatic enteritis associated with *Clostridium difficile*, 5 patients (1.9%) experienced rashes, 1 patient (0.4%) suffered from joint pain, and 2 patients (0.8%) reported fatigue. All symptoms improved with treatment, and no serious adverse events were found. Additionally, there were no cases of medication discontinuation due to serious adverse events.

## Discussion

In this multicenter cohort study, we investigated the real-world clinical and objective effectiveness of VDZ in Chinese CD patients in a real-world setting. Our findings indicate that VDZ is efficacious in both induction and maintenance phases, with a favorable safety profile. Notably, bio-naive patients in remission or with mild activity (CDAI ≤ 220 points and CRP < 10 mg/L) at baseline, without active intestinal fistula, showed the highest probability of positive response to VDZ. These insights contribute to refining patient selection for VDZ treatment in the clinical management of CD.

Real-world studies can reflect the efficacy of drugs in practical settings, since a considerable portion of patients may not meet the criteria of randomized, placebo-controlled clinical trials. Since the approval of VDZ, several real-world cohorts of patients treated with this drug have been studied, and their efficacy and safety results have been published [21–27]. A meta-analysis including 13,663 CD patients showed that the pooled estimate rates of clinical remission were 36% at induction and 39% at maintenance phases [28]. A cohort study conducted in Scotland showed that the 12-month cumulative rate of mucosal healing was 38.9%. In contrast, another cohort from Canada showed that the objective remission occurred in 21.2% and 18.9% of patients at 6 months and 12 months, respectively [29,30]. Objective results were equally important, as we know clinical symptoms are poorly correlated with the degree of intestinal inflammation in CD, which was not uncommon to discover intestinal inflammation despite patients being in clinical remission [31]. To more accurately assess the level of inflammation activity, we utilized objective evaluations such as endoscopy, ultrasound, and radiology, and the outcome definitions of these evaluations have been widely used in other studies [15,32,33]. In our cohort, 36.8% of patients were in clinical remission at baseline, but nearly 60% of them were in the objective active stage. So we included a population receiving VDZ treatment for various reasons and reported the efficacy of VDZ from different efficacy endpoints, which may reflect the therapeutic effects more objectively. We found that the rate of steroid-free remission was 47% and 39% at Weeks 26 and 52, respectively, which were consistent with the previous studies. The rate of objective remission was also comparable with that of the Western population. Notably, a considerable portion of the study population experienced a high inflammatory burden (e.g. more than 50% of patients were in stricturing and penetrating phenotypes, and 15% of patients had active fistulas at baseline), leading to the low objective remission rate. The persistent objective inflammatory activity may affect physicians’ decision-making regarding alternative therapy without dose optimization, despite a small proportion of patients still being in the clinical remission stage. This may be one of the possible explanations for the lower efficacy and higher drug discontinuation in the long-term stage.

For CD patients, there is a significant need for a reliable prediction model to guide future therapy, instead of using a one-size-fits-all approach [34]. The VDZ-CDST developed by Dulai *et al.* has the highest quality of evidence currently [9,35]. The VDZ-CDST consisted of 5 parts, and patients with a high CDST score (no prior bowel surgery, no prior tumor necrosis factor-α antagonist, no prior fistulizing disease, adequate albumin, and low CRP concentration) showed the highest probability of responding to VDZ. The CDST has been validated in the VICTORY consortium, with an AUC of 0.66 in predicting steroid-free remission and 0.72 in predicting mucosal healing at Week 26 [9]. However, the clinical application of the scoring system may be limited due to its complexity in calculation, and its effectiveness in a real-world setting needs to be determined. In our study, we identified several risk factors in predicting non-response to VDZ in a real-world setting. We found that patients who were in remission or mild activity at baseline, determined by CDAI (≤ 220 points), and did not have active intestinal fistula (rather than prior fistulizing behavior) could predict the effectiveness of VDZ more accurately, as these two factors may be considered as exclusion criteria for Phase III clinical trials but are common in real-world practice. We stratified the overall population based on the number of risk factors, which allows for easier clinical application. Our findings indicated that patients with no mentioned risk factors showed a high probability of responding positively to VDZ in clinical and objective measures both in induction and maintenance phases, and exhibited a high rate of drug continuation. We also developed a nomogram model using the aforementioned risk factors for further confirmation, with the AUC ranging from 0.693 to 0.830, indicating a good predictability in our cohort. These factors can be valuable in identifying high responders in real-world practice.

In our cohort, the safety profile of VDZ was consistent with observations from clinical trials and two extensive safety studies [36,37]. Mild allergic reactions and fatigue constituted the most common infectious adverse events, and a few infusion reactions were observed. Although serious adverse events were not found in our study, 1.1% of patients developed symptomatic enteritis associated with *Clostridium difficile*, which warrants attention.

Our study has several strengths. To the best of our knowledge, this is the largest real-world cohort investigation on the effectiveness of VDZ in both bio-naive and bio-exposed patients with CD in the Asian region. The comprehensive clinical and objective effectiveness data contribute to its credibility. However, it is important to acknowledge several limitations. First, the retrospective observational design, despite efforts to standardize definitions and establish consensus, poses inherent constraints. Second, the predominant inclusion of patients with mild-moderate disease activity at baseline may limit generalizability to severe CD cases. Third, ileocolonoscopy (SES-CD) and cross-sectional imaging were employed in accordance with STRIDE recommendations to assess ileocolonic disease activity [14,38]. However, a key limitation is the potential underestimation of inflammation in the proximal small bowel. This challenge in reliably quantifying disease activity beyond the terminal ileum with current standard modalities is a well-recognized limitation in the clinical evaluation of CD, not unique to our study. Our findings, therefore, should be interpreted within the context of this inherent constraint of existing assessment tools, particularly in patients with proximal small bowel involvement. Finally, the risk factor-based population stratification derived from multivariate analysis is confined to our retrospective cohort, necessitating validation through external prospective real-world cohorts.

## Conclusions

In this multicenter real-world study, we demonstrated the clinical and objective effectiveness of VDZ in Chinese patients with CD. Our findings indicate that VDZ is effective in both inducing and maintaining steroid-free remission in CD patients. Notably, VDZ demonstrated superior outcomes in bio-naïve patients who did not have active intestinal fistulas and exhibited a low inflammatory burden. Though these results are promising, it is essential to validate them through larger-scale prospective studies to provide conclusive evidence.

## Supplementary Material

goaf096_Supplementary_Data

## Data Availability

Data may be available from the corresponding author upon reasonable request.
