# Supplementary material for: Real-World Effectiveness and Patient Stratification for Vedolizumab Treatment in Crohn’s Disease: A Multicenter Retrospective Study
Source: Gastroenterol Rep (Oxf). 2025 Oct 31;13:goaf096. doi: 10.1093/gastro/goaf096 (PMC12576324; doi:10.1093/gastro/goaf096)
Supplement: goaf096_Supplementary_Data [file goaf096_supplementary_data.zip › supplementary_materials final 20251011.docx]

**Supplementary Materials**

**Supplementary Table 1.** Coefficients of predictors in LASSO and multivariate regression analyses.

| LASSO regression (λ.min*) | |  | Multivariate regression analysis | | |
| --- | --- | --- | --- | --- | --- |
| Predictor | Coefficient |  | OR | 95% CI | *P* value |
| Intercept | 0.9202 |  |  |  |  |
| Active intestinal fistula | -0.8907 |  | 8.345 | 2.469–28.206 | 0.001 |
| Bio-exposed | -0.6632 |  | 3.646 | 1.925–6.906 | < 0.001 |
| EEN-exposed | -0.0855 |  | 1.879 | 0.980–3.600 | 0.057 |
| Steroid-exposed | -0.0582 |  | 1.543 | 0.802–2.970 | 0.194 |
| CDAI > 220 points | -1.3263 |  | 8.528 | 4.135–17.591 | < 0.001 |
| CRP > 10 mg/L | -0.2527 |  | 1.969 | 1.014–3.821 | 0.045 |
| Female | 0 |  |  |  |  |
| Age | 0 |  |  |  |  |
| Disease duration > 24 months | 0 |  |  |  |  |
| Active smokers | 0 |  |  |  |  |
| Disease location L2 | 0 |  |  |  |  |
| Disease location L3 | 0 |  |  |  |  |
| Disease behavior B2 | 0 |  |  |  |  |
| Disease behavior B3 | 0 |  |  |  |  |
| Active perianal disease | 0 |  |  |  |  |
| Previous immunomodulators | 0 |  |  |  |  |
| ALB < 35 g/L | 0 |  |  |  |  |

*Coefficients estimated from LASSO regression at λ.min = 0.054 (10-fold CV); ALB = albumin, B2 = stricturing, B3 = penetrating, CDAI = Crohn’s disease activity index, CI = confidence interval, OR = odds ratio, CRP = C-reactive protein, L2 = colon, L3 = ileocolon, LASSO = Least Absolute Shrinkage and Selection Operator, EEN = exclusive enteral nutrition.

**Supplementary Figure Legend**

**Supplementary Figure 1**. Calibration curves and decision curve analysis curves of the nomogram model.
