# Supplementary figures and images for: Real-World Effectiveness and Patient Stratification for Vedolizumab Treatment in Crohn’s Disease: A Multicenter Retrospective Study
Source: Gastroenterol Rep (Oxf). 2025 Oct 31;13:goaf096. doi: 10.1093/gastro/goaf096 (PMC12576324; doi:10.1093/gastro/goaf096)

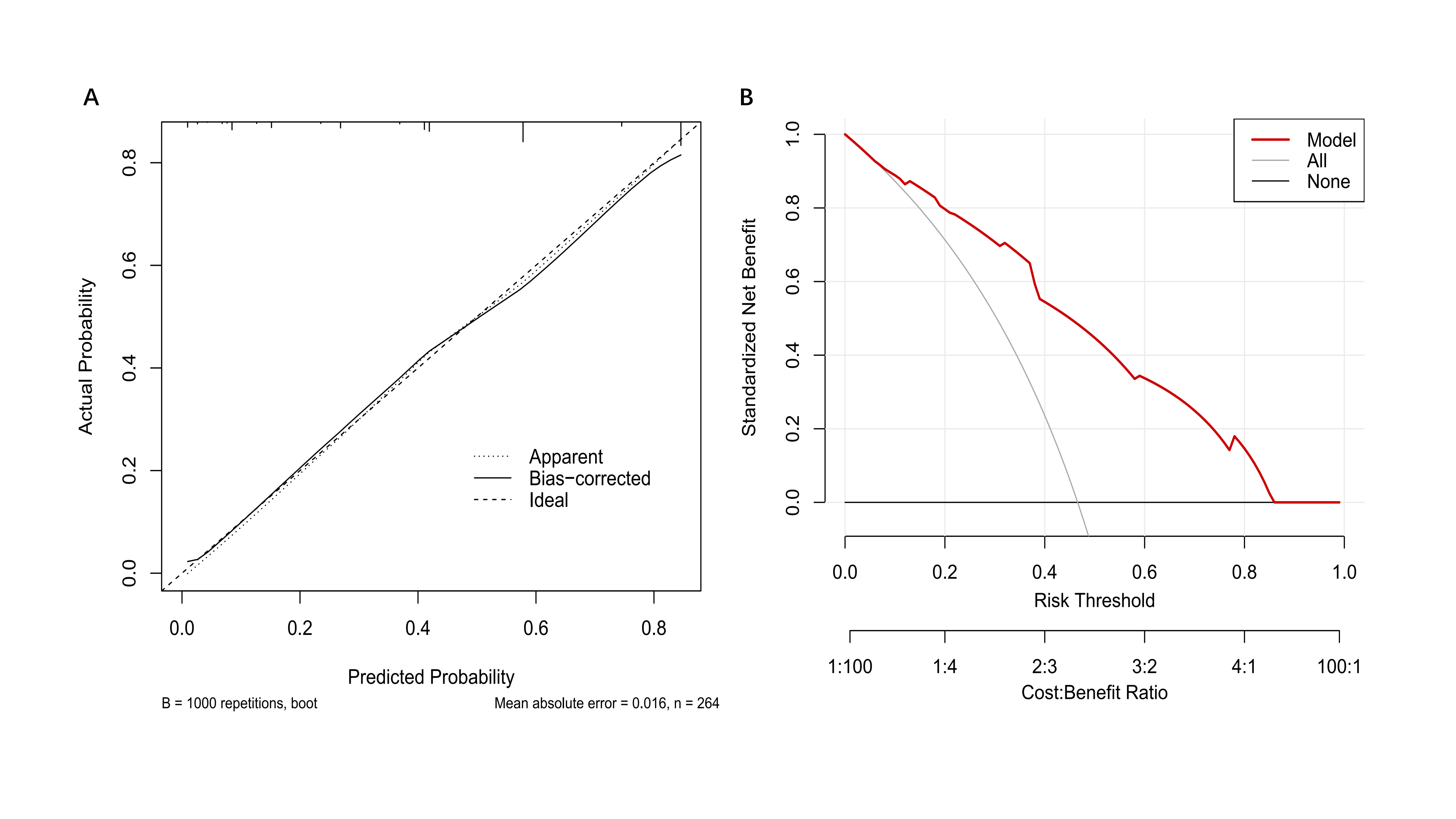

Supplement: goaf096_Supplementary_Data [file goaf096_supplementary_data.zip › supplementary figure 1.TIF]
